# Supplementary material for: Optimal algorithms for controlling infectious diseases in real time using noisy infection data
Source: PLoS Comput Biol. 2025 Sep 3;21(9):e1013426. doi: 10.1371/journal.pcbi.1013426 (PMC12478939; doi:10.1371/journal.pcbi.1013426)
Supplement: Attachment — Submitted filename: Response to reviewers.pdf [file pcbi.1013426.s004.pdf]

Responses to Reviewers: PCOMPBIOL-D-24-01328

We thank the reviewers and editor for their constructive and insightful comments. We respond to every point and outline corresponding changes to the main text below. We also include both clean and tracked-change versions of the new text to highlight our revisions. References made here are listed in a bibliography at the end of this document.

Editor

A point of my own is that you only analyse the effect of an overall restriction effect, and do not compare different preventive measures. When I read the abstract and introduction my feeling was that you also studied cost-benefit effects of different preventions, so clarify better that this is not the case.

Thank you for this comment. While we do not examine the different and specific mechanisms by which interventions are implemented, we do still compare measures in terms of their effect on the effective reproduction number, R, their future-discounted, accumulating costs and the toll of the resulting trajectory of infections under those measures. This is because we only consider semi-mechanistic (renewal) models. Our approach roughly mirrors tiered intervention policies, i.e. we decide among intervention packages of differing stringencies based on their expected benefits and relative costs. Highly impactful studies such as [1], [2], [3] *retrospectively* modelled numerous interventions, using analogous renewal models, via their multiplicative effect on R. Such works and their estimated R-effects form the background and context for our approach.

Our model combines those effects with costs and other benefits (e.g., peak size or endemic load) to make decisions *prospectively* about which among the suite of possible interventions should be initiated or removed at any given policy review time. Comparison is therefore in terms of R, costs (or proxies for costs) and the projected epidemic trajectories under the interventions in our suite. Provided we know an intervention's reproduction number effect (cR_0, with c as the effect) and cost (extracted from studies like [3]), we can always include it within this suite (or in our action space). We have added clarifying comments in the Abstract, Author Summary and Introduction that explain these points and further distinguish our approach from more mechanistic intervention evaluations. Hopefully, this now prevents any confusion and is more representative of our work.

Reviewer #1

The paper reads well and shows how feedback control can be used to minimise the societal costs during the early months of a pandemic. This is an important problem, and I am glad that different scientists approach it in different ways.

Thanks for the positive comments. We are pleased that you found merit in our feedback approach.

1) In terms of the literature cited, I think that the authors could include a reference to Morris, D.H., Rossine, F.W., Plotkin, J.B. et al. "Optimal, near-optimal, and robust epidemic control". Commun Phys 4, 78 (2021)[]. The authors main intuition is also similar to that of Di Lauro, Kiss, Rus and Della Santina "Covid-19 and Flattening the Curve: A Feedback Control Perspective," in IEEE Control Systems Letters, vol. 5, no. 4, pp. 1435-1440.

I noticed that there are some differences in terms of which models have been uses for the epidemic, and how to define the optimal control. The authors could probably add some discussion on how their results are different or novel with respect to this paper.

Thank you for these suggestions, we now refer to both papers, [4] and [5] here, in the manuscript. Compared to previous optimal control efforts, our paper presents several novelties pertaining to the treatment of stochasticity, practical surveillance uncertainties and choices of control signals. The epidemic feedback algorithms that we develop and apply are also a contribution to the field.

In terms of methodology, instead of the deterministic SIR model in [4] and the mixed approach in [5] (they consider stochasticity in simulating the epidemic but use a deterministic surrogate for informing control) we use the stochastic renewal model which considers the randomness intrinsic to disease transmission [6] and is known to generalise the dynamics of SEIR and related models [7]. A benefit of our approach is that while it is flexible and widely used to model various diseases (e.g., Ebola virus disease, COVID-19, SARS, measles, influenza) it only requires knowledge of the reproduction number and generation time distribution, which are commonly estimated during outbreaks [1], [3]. Importantly, this model is frequently used to inform real-time response [8].

Another key distinction lies in the control signal we use to trigger action. Much of the literature, including [4], [5], focuses on prevalence-based control. However, estimating prevalence is difficult and often requires extensive testing programmes, e.g., the REACT programme in the UK [9]. We frame our optimisation in terms of the incidence of new cases, aligning more closely with real-time policy triggers obtained from routinely collected and updating data. We also highlight that the critical issue of surveillance noise, particularly reporting delays and case under-reporting, is only minimally addressed within [4], [5], whereas our research places a strong emphasis on this aspect and its important but understudied consequences for control.

Further, the control questions examined in these studies are different from each other and our work. Specifically, [4] investigates a single "one-shot" intervention of fixed stringency where the beginning end times are optimised. In contrast both [5] and our paper consider continuous-time interventions based on a target and investigate various practical limitations of real-time epidemic control, e.g. discrete actions space and policy review times. However, there are several crucial distinctions between how [5] and our work proceed and in what findings consequently emerge.

First, control objectives differ fundamentally. Our interventions adapt continually to the epidemic's realised trajectory, whereas [5] attempts to steer the system along a single pre-computed optimal path. Second, the modelling assumptions in [5] omit several real-world complications that we address. Particularly, [5] assumes full case ascertainment, does not account for intervention costs, focuses solely on health outcomes and does not investigate the effect of dispersion in reporting or disease-progression delays. Our framework resolves these gaps. The novel algorithms that we develop weigh epidemiological benefits against both economic and social costs and our results reveal the impact of realistic distributed delays and stochastic under-reporting. We optimise public health targets and costs simultaneously and in real time, whilst re-estimating important epidemic parameters, thus being able to react to any changes in epidemic dynamics as they unfold.

The focus of our study and results also differ from [5], where the authors main aim is to provide validation of their method on realistic simulations. While realistic validation is among our goals, our focus is on exploring and exposing the largely unknown limits imposed by surveillance noise when designing real-time interventions. This caused ongoing debate around the value of closed versus open-loop control strategies. Our study is the first (as far as we are aware) to benchmark what feedback-based methods can achieve for infectious disease response by devising a model predictive control algorithm that factors in the complexities of transmission and surveillance and showing how and when it outperforms strategies that have been used in policy (threshold-based or event-triggered approaches) or have been proposed to be more robust to noise (time-triggered approaches). Such extensive analyses of the effect of surveillance noise on control performance has not been done in the literature before.

We also demonstrate key robustness properties of our algorithm by simulating scenarios when the MPC algorithm must face unmodelled transmission dynamics or tolerate uncertainties in NPI efficacies. These yield novel results and insights into what is achievable in real time. Neither [4][5] assess performance under unanticipated epidemic dynamics. We have now edited the Introduction to reflect on some of these key differences and better contextualise our contributions.

2) I also wonder if the authors can prove mathematically that their control strategy is optimal in some sense. Why the reward function (4) is better than different choices? What is the effect of different rewards?

This is an interesting point. However, mathematically proving global optimality under stochastic dynamics that are non-Gaussian is inherently challenging and would be a separate paper on its own (and is out of scope of this study). In designing our model predictive controller we employed a Receding Horizon Control approach, i.e. we choose the NPI with the optimal reward projected across our moving time-horizon. This method is widely used in control theory to handle real-time adjustments in a dynamic system see, for example in [10] and [10].

At each policy revision, we choose the action minimising the discounted total cost for our projection horizon. This strategy has two key important advantages over planning across the entire epidemic. First, evaluating long sequences of decisions quickly becomes intractable since computation time grows exponentially with the number of possible actions. Second, long-range plans are fragile; they ignore increasing uncertainty in extended projections, which we also account for by applying discount weight factors.

This means that, by design, the MPC approach optimises interventions over a short time horizon rather than an infinite one (or over the entire timeframe of the epidemic outbreak). Nevertheless, classical results in (receding-horizon) model predictive control show that, with an appropriately designed cost function and constraints, the receding horizon controller is provably stabilising and approaches the infinite-horizon optimal solution as the horizon length increases [11].

However, given the issue of exponentially increasing computational burden, we cannot increase our projection horizon indefinitely. Instead, we tackle the issue of long-term optimality by restricting the controller to only consider the first action to be taken and optimise the projection horizon within these constraints. This is done as part of hyperparameter tuning (including other

hyperparameters such as the cost discount factor). For this, we employ Bayesian optimisation on our control parameters. Here, we optimise the same cost function as the one used within the MPC algorithm evaluated over a longer period (31 weeks) with daily costs weighted equally (no discounting) to account for actual performance over the timeframe of an epidemic. This approach enables us to achieve near-optimal performance in steady state with the receding horizon approach whilst still preserving the advantages of this method's adaptability to changes in epidemic dynamics. As an example, in Fig. 1 below we plot our optimisation for the MPC horizon length. The optimiser identifies a 12-day projection horizon as best for controlling Ebola virus disease. For our COVID-19 simulations the optimal receding horizon was also 12 days.

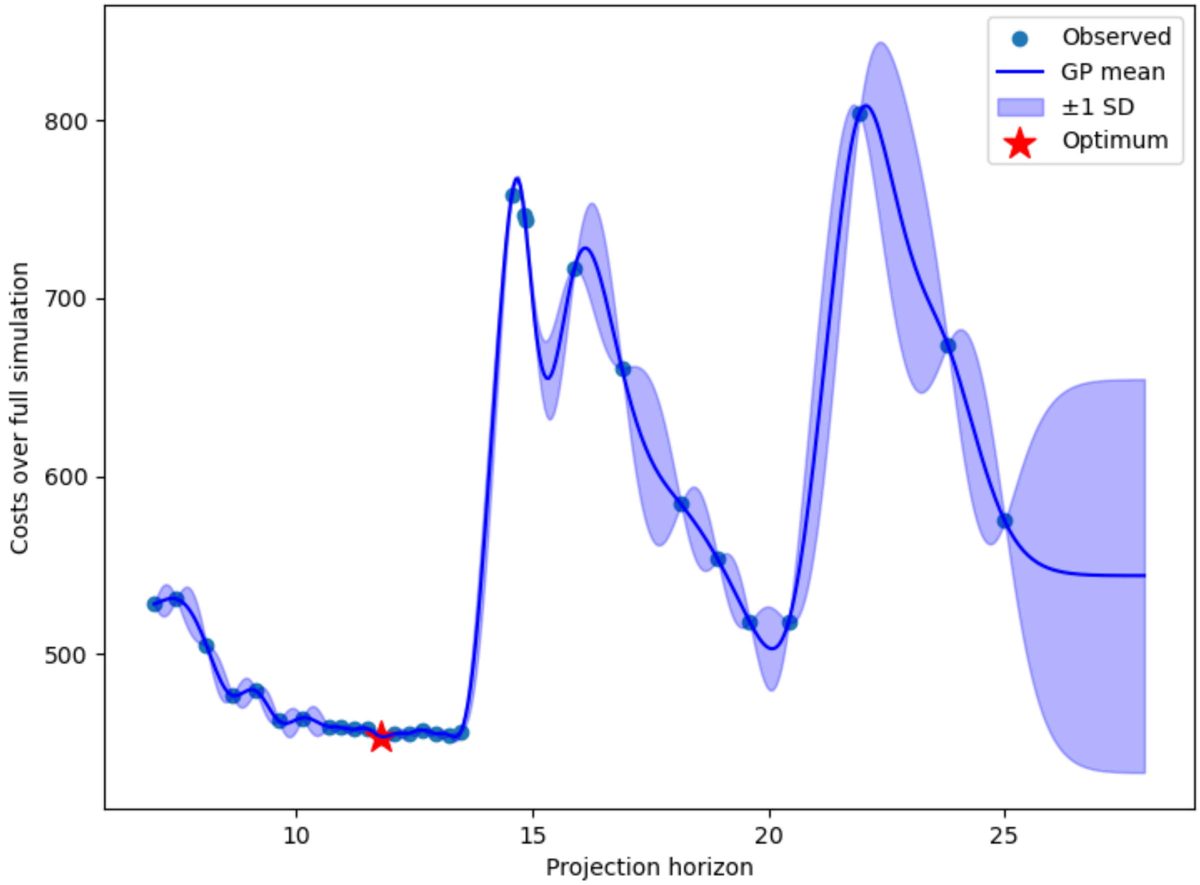

Figure 1. Bayesian optimisation of projection time-horizon for controlling Ebola Virus Disease with a policy review period of 7 days and a simulation length of 31 weeks. The blue markers indicate function evaluations, while the blue curve and band show the fitted Gaussian Process model that is employed within the Bayesian optimisation algorithm. The red star indicates the optimum identified by the model, indicating that a receding horizon of length of 12 days provides the best performance.

Although Bayesian optimisation does not guarantee a global optimum, it does efficiently balance exploration and exploitation, helping us escape local minima with far fewer function evaluations. Our baseline results (without surveillance noise) show that the MPC algorithm with the identified parameters is effective for keeping the daily infection incidence near the target level.

Our reward function (Equation 4) represents a specific set of cost-benefit trade-offs, which is less common in epidemiological models (which frequently emphasise reducing transmission) but not as extensive as health economic models. This balance allows us to guide real-time policies and include important disease stochasticity. Our reward formulae explicitly encode targeted objectives. This choice is pragmatic: it allows policymakers to gauge, within a unified scalar objective, how much economic activity would be traded to reach desired health outcomes. This objective is, of course, not unique. For example, if one prefers to minimise the squared deviation of incidence from some target level, then large overshoots would be penalised more harshly, and the resulting intervention schedule would differ. Thus, we do not claim that our reward function is universally "better" but rather that it is reflective of priorities that are central to our study and solutions will undoubtedly depend on the choice of the reward function. As an illustration, in the extreme case that we do only care about economic output (neglecting health effects), then "no intervention" will be always selected as the (trivial) optimal policy. We also highlight that our MPC algorithm is flexible to optimise NPIs with alternative cost functions and target objectives (e.g. [12], [13]).

There are also alternative ways of deciding how to balance intervention risks and costs. These often result in the solving of different control problems. For example, in [14] economic output is maximised, with health "targets" used as optimisation constraints, while [15] purely focuses on minimising peak incidence or total number of infections over a period of interest[15]. Our study does not assess or challenge these other design choices. Policymakers may express different targets in different contexts (or e.g., in different locations even when the data are similar). We believe our approach is a valuable addition to the literature and useful because both our cost functions and control action space can be customised to the specific and strategic priorities of policymakers and modellers. In some cases, as appropriate costs and efficacies may not be known, our algorithm also allows rapid exploration of various hypothesised scenarios, narrowing the search space to identify the most useful reward formulations for real-time decision-making.

3) As the agent response depends on his ability to predict the number of infections after t days, what is the impact of model error? For instance, if a new variant arises, or if new evidence builds up showing that there are multiple routes of transmission between hosts, and therefore the estimates for the transmissibility of the pathogen change?

Our paper focuses on how data errors, particularly under-ascertainment and reporting delay, impact control outcomes. We parameterise the model with the best available knowledge for the generation time distribution and the basic reproduction number. Although deeper model errors (e.g., those arising from new variants or additional transmission routes) lie beyond our present scope, our MPC algorithm is robust to changes in epidemic dynamics because it recalculates R_t from recent case data at each decision timepoint. This feedback control feature helps correct for evolving conditions, such as variant emergence or uncertainties in the efficacies of the NPIs. We present examples of this adaptability in Fig. 7 and a new Results subsection, showing the ability of our MPC approach to tolerate the resulting and unexpected transmissibility changes. Further, because our receding horizon is short and we frequently re-estimate the reproduction number, model error is limited in how much it can accumulate and there is inbuilt robustness.

Of course, robustness has its limits and if a change is so dramatic that our strongest NPI is unable to ever bring the effective reproduction number below 1 then our control algorithm will not be able to stabilise the epidemic (e.g., this may mirror what happened when Omicron emerged during the COVID-19 pandemic). However, in that case the failure of our algorithm would quickly indicate that more stringent measures are needed, hence still providing useful information. Moreover, our approach is synergistic with others as if better forecasting methods exist (e.g., from forecasting hubs [16], [17]), we can directly include these forecasts within our algorithm to improve control performance. We have included comments on these points in the Discussion.

I recommend minor revision based on these observations.

Reviewer #2: This paper represents an interesting addition to the literature. I have a number of major comments:

1) Structure of the paper and length: I do not think that the current structure of the paper (Introduction-Results-Discussion-Methods) is the most appropriate for this article. Authors make some efforts to explain the methods in the results but it remains difficult from these explanations to understand what is being done without reading the Methods section. The methodological explanations provided in the Results section (and repeated in the Methods) also substantially increase the length of the manuscript, which is currently too long. I feel it would be much better if methods section was moved to before the results section. Authors should ensure all methodological descriptions are put there and remove the many methodological descriptions from the results section. Results section should only present the results. I feel these change would substantially improve the experience of the readers. Shortening the manuscript will also improve this experience.

We have restructured the manuscript as suggested (the Methods section is now before Results and redundant content has been removed). The reason we had placed the Methods last originally was to match conventions from the infectious disease literature. We prefer having the Methods before Results and agree that this formatting improves the clarity and conciseness of the paper.

2) Uncertainty about the impact of interventions "Although in reality the factor ct is unknown and needs to be estimated, we assume throughout this study, that the effect of any NPI on the reproduction number is known and without uncertainty." I think that this is a key limitation of the current work. In practice, the impact of an intervention is never perfectly known. This would impede the relevance of this framework in real-world situations. Authors briefly explain the issue of uncertainty in Supplement Figure S9 but explanations about what is done are lacking. This issue receives very little attention in the manuscript. Given that this is a critical aspect, potentially with key impact on relevance of method in real epidemics, I strongly suggest that this question receives more attention in the main manuscript and is more extensively discussed.

We agree with the reviewer that uncertainty in NPI efficacy is important but had not discussed this extensively because the focus of this study was on how uncertainty from surveillance data (observation noise) limits feedback control. In order to isolate and better quantify the effect of this observation noise, we did not add uncertainty in cR0 to most simulations, assuming that c was

either well known or chosen to test possible scenarios (e.g., often modellers set and test various c options when supporting intervention decisions). Our goal was to expose the fundamental limits induced by practical epidemic surveillance, revealing key insights into how, even under perfect knowledge of interventions, delays and ascertainment levels critically restrict controllability.

However, we do acknowledge that uncertainty in NPI efficacy is an important factor that affects policymaking and we now better highlight that our control framework is able to model this effect. We add explanatory text in Discussion and reposition the supplement S9 figure as the new Fig 7 of the main text (within a new Results subsection) to emphasise this result. In the caption and discussion of this figure we add more details on our simulation and on how our control algorithm still performs well amidst uncertainty in the impact of NPIs (i.e., we introduce Beta distributions over possible c values, which affect projected costs and benefits). We also include a paragraph on the criticality of this uncertainty in the Discussion and how we can input estimates from the literature directly into our algorithm to handle and ameliorate the impact of this uncertainty. For example, we can use c estimates (with uncertainty) from [1], [2], [3] to ensure our projections are as accurate as possible and hence capable of informing real-time policy decisions reliably.

3) Correcting for underreporting or reporting delays: In simulation studies, authors explore the impact of under-reporting and reporting delays. However, it is unclear whether what they do is consistent with what analysts would do in this real epidemics. For example, when analysing an epidemic in real-time, analysts would typically be aware that there is a reporting delay; and correct for it. Here, if I understood correctly, authors do not make any correction (?). To me, this means that the results they present in scenarios with under-reporting and reporting delays may be too pessimistic. What would the results be like if modellers were making corrections in real-time (which they would do in practice)? It is wrong to say that we are not aware of these problems when they occur. There may be uncertainty about the exact values of the reporting parameters but we usually have a pretty good idea of possible range. This should at least be discussed.

Thank you for this thoughtful comment. The reviewer is right that, in real-time epidemic analysis, reporting delays and underreporting are typically recognised and, where possible, corrected. Our aim here, however, was not to simulate exactly what analysts should or would do in real-time, but to understand the intrinsic limitations that common imperfections in data impose on intervention decisions. Consequently, in our simulations, we intentionally did not apply delay or underreporting corrections. We also compared against a baseline of no noise (perfect surveillance) to reveal what bounds possible control performance (these limits are unknown as far as we can tell).

Our results represent performance across a wide spectrum of noise levels reflecting the range between a naive approach and the ideal of perfect information, rather than making assumptions about how well corrections could be applied in practice. No matter what correction is applied (and we note, for example, that during the initial stages of the COVID-19 pandemic some corrections actually induced biases [18]) realisable performance should sit somewhere within these bounds. We now outline these points in the Discussion. We also highlight that corrections can quickly become irrelevant or outdated as recently emphasised in [19]. Our work by defining best (no

noise) and various worst-case limits (differing noise levels) provides a reference for benchmarking the types of performance we might expect during an unfolding outbreak.

We also highlight that surveillance quality varies substantially across contexts. In some settings, e.g. in countries where the surveillance system is underdeveloped, noise correction may not be feasible due to a lack of reliable metadata on delays or underreporting [20]. Correction methods also rely on strong assumptions (e.g. assumed or badly inferred delay distributions [21]), which may only hold under specific conditions and may not generalise across settings or times. Further, even if corrections are applied, policy implementation is subject to additional delays, e.g. due to review periods, or time from decision to announcement and implementation, which may hinder timely response. These factors will make any correction method inherently imperfect, and as a result, they may reduce but not eliminate noise and uncertainty in data.

We therefore believe that there is value in understanding worst-case and best-case scenarios, particularly for planning and identifying the kinds of noise that most impair epidemic control. We also observe that the field of correcting for surveillance imperfections is very active [18], [22], [23] indicating this is far from a solved problem. We do note that our framework could be extended to incorporate inferred infection time series (e.g. using methods that reconstruct true infections from observed data [24]) to assess how well control performs under such corrections. In this application, simulation frameworks like ours could play an important role in testing noise correction methods (e.g., we could flag if a correction derived from some auxiliary data type improves controllability – if it does not this might evidence that resources are better allocated to collecting other data types). We include several of these points now in the Discussion.

4) Figure 2 (and other similar figures): Figure 2 and similar figures are very hard to navigate. They are central to understanding what is happening so it is important that they are substantially improved. Specifically, I find it difficult to read/understand the different colored curves in Figure 2. I find the formulation “New cases/infections” confusing in Figure 2C. Difficult to navigate which is which. I cannot easily assess which is the thick highlighted curve and the thin highlighted curve…Please improve presentation/readability. It might be easier to understand the Figure if there was 1 panel for infections and 1 for cases.

Thank you for the constructive feedback on the figures. We improved the readability in Figs. 2, 5 and 8 (re-numbered now as 3, 6 and 7) showing simulation results by implementing several changes. First, in the left panels, we use the colour codes for NPIs only for the thick curves representing the reported cases in the highlighted realisation. The corresponding true incidence curve (where different) is shown in blue. This colouring convention now also matches panel (d) in Fig. 1, explaining the different types of surveillance noise. Here, the reported cases are show in black (without NPIs), and the incidence curve is shown in blue.

Second, in the right panels, we only use the colour codes for the effective reproduction number whereas the reproduction number estimated from cases are shown in orange uniformly. Third, we now uniformly changed our labels to “New cases” and highlight in the caption that we show the true infection incidence only for the highlighted realisation in blue. We also note that in rows (a)

and (d) in Figure (3), we do not show a separate “true infections” curve as this is identical to the reported cases in scenarios featuring no surveillance noise.

5) Authors consider a model where there is no depletion of susceptible, even though [25]epidemics appear to be quite large. Are we sure that ignoring the depletion of susceptibles is not a problem in the simulation scenarios authors are looking at? What is the proportion of the population infected by the end of the simulations? Please discuss.

Thank you for this remark. It is correct that in our simulations we do not constrain the size of the host population and susceptible depletion is ignored. While it is reasonable to question whether this simplification is problematic or realistic, this was a deliberate modelling choice to ensure that we can best extract signals about control quality. We note that similar renewal models that neglect susceptible depletion were widely used at the national level during the COVID-19 pandemic, often yielding incidence estimates of roughly 100,000 new infections per day [1], [26], [25]. This strategy of not explicitly tracking susceptible depletion, was common when inferring reproduction numbers, evaluating the impact of interventions and ascertaining the transmission advantage of emerging pathogen variants [27]. These precedents support applying our algorithm at this scale to inform intervention decisions.

We chose not to explicitly include susceptible depletion because we are controlling to a specific target and are primarily interested in control performance, which is measured by peak size and the steady-state envelope. If we included susceptible depletion, even poor control strategies might appear to perform well, simply because the epidemic would eventually self-limit through herd immunity, masking the true performance of the control algorithm. Without susceptible depletion, the model gives a clearer picture of how well the control strategy itself is working.

We highlight however, that our MPC algorithm is adaptive and robust. It recomputes the effective reproduction number R from data at each decision point. Although the underlying model does not explicitly track susceptible depletion, this estimated R inherently reflects its influence, because changes in transmission, whether from behaviour, immunity, or depletion, are implicitly embedded into the observed R. Further, the control algorithm only uses short-term projections. Over these timeframes, susceptible depletion has minimal impact on dynamics and so would not affect the decision-making or optimisation process. If it did start to affect outcomes significantly, that would be picked up in the next data update and automatically incorporated into the algorithm’s response.

To evidence our comments, we have included a simulation below that does include depletion of susceptibles. It demonstrates that the envelope eventually collapses as herd immunity is reached. This confirms that metrics like peak size and envelope no longer reflect control quality when susceptible depletion dominates, which is why we exclude this effect from this study. However, this simulation does confirm that our algorithm can easily include depletion if required to answer other questions beyond the scope of this work such as how best to combine NPIs with depletion.

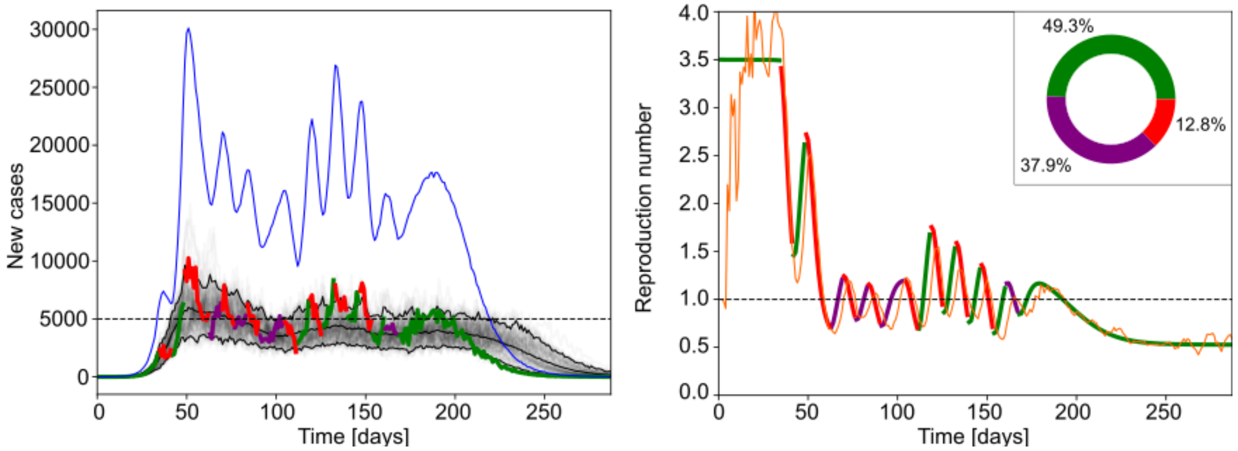

Figure 2, Optimal epidemic control for realistic simulated epidemics with surveillance noise (delay – mean: 10.5, dispersion parameter: 50.0, reporting-rate: mean: 0.3, a = 8.0) with susceptible depletion in a population of 1 million. As immunity builds up in the population, the MPC algorithm keeps new cases around the target by gradually applying less stringent actions. After reaching herd immunity, no further restrictions are applied.

6) Page 2 "Other works have focussed on optimising the timing of specific interventions considering a 'one-shot' control with the start and ending time to be optimised ». Other relevant references that could be added in this paragraph include Tran Kiem et al, Eurosurveillance "Lockdown as a last resort option in case of COVID-19 epidemic rebound: a modelling study" 2021

We have now added a reference to Tran Kiem et al. (2021) Eurosurveillance, which is a relevant example of work optimising the timing of a one-shot intervention.

7) Figure 4: I don't think presenting results as a function of parameter "a" is very helpful. Please present with respect to a parameter that is easier to interpret than a.

We agree that the dispersion parameters could be better explained. To aid the reader, we added a new figure to the supplement showing all the delay and reporting rate distributions used in our analysis to clarify the use of the dispersion parameter.

8) Discussion p11: "However, the time-triggered strategy also has notable drawbacks because its design requires precise knowledge of both the epidemic parameters and the efficacy of each NPI. Consequently, this strategy is still implicitly linked to infection incidence data in practice. We therefore find strong evidence that the additional complexity, relative to reference strategies, such as event and time-triggered approaches, involved in performing MPC brings substantial advantages » But MPC also requires knowledge of impact of interventions, which is often far from perfect and has usually to be estimated from the data being analysed… See point above

Thank you for your comment. To clarify, we do not claim that MPC does not require knowledge of the epidemic state or the likely impact of interventions. Instead, our point is that time-triggered strategies, although open-loop by design, are much more heavily reliant on having accurate prior

knowledge of epidemic parameters and NPI efficacies. Consequently, time-triggered approaches become extremely non-robust to changes and uncertainties (e.g., if transmissibility is different from what is expected). MPC does not share this lack of robustness.

The reason for this distinction is that MPC incorporates feedback; it can adjust the control strategy dynamically as new data emerge, which makes it more robust to model misspecification, timing errors, or changes in epidemic dynamics. We illustrated this capacity in Figure 7 which shows how MPC maintains good performance under significant uncertainty. The only relative but small weakness of the MPC algorithm is that under extreme delays in case-reporting, mistimed-action can drive sub-optimal control. In contrast, because the time-triggered method acts at the same preset time under all circumstances, it still performs reasonably in this extreme scenario. However, our study demonstrates that under moderate and realistic noise levels, MPC outperforms the time-triggered approach considerably, while still providing important robustness properties. We edited the paragraph referenced above to reflect these points and clarify our claims.

9) P12: "For example, if immunity is acquired by infection, then this reduces the susceptible population and decreases the effective reproduction number. In our modelling framework, this can be easily included by setting Rt = R0tS/N, where S and N are the susceptible and the total population, respectively. Note that we excluded this from our simulations in order to isolate the impact of the NPIs and to allow fair comparison among epidemics that have dynamics on differing timescales." But S not monitored? Underreporting not accounted for.

With respect to susceptible depletion, please refer to our response to Question 5) above. We agree that monitoring the susceptible population S in practice is challenging, particularly due to the underreporting of infections. However, the MPC algorithm does not explicitly incorporate susceptible depletion. Since the projections used to determine optimal control actions are short-term, this simplification does not induce large errors. If anything, it may lead to conservative estimates, which is preferable from a public health perspective.

In the paragraph the reviewer referenced, we simply noted that susceptible depletion could be included in the simulation framework by adjusting the ground truth model (i.e., the system that generates the epidemic data), without changing the structure of the MPC algorithm itself. The MPC framework would still function as intended, as changes in susceptibility would be reflected indirectly through the estimated reproduction number R_t.

We also acknowledge that we did not specify how S was calculated in the simulation example. This has now been clarified as S = N - \sum I, which is a common method for including susceptibles in renewal models [1], [28]. However, this does not affect the MPC algorithm's operation, as its only link to susceptible depletion is implicit, via inferred changes in R_t. This parameter naturally declines as immunity builds through infection. Last, we note that difficulties in monitoring S are a general problem in the field and not an issue specific to our algorithm.

10) Page 13: "Our MPC algorithm is also capable of accommodating uncertainty in the efficacy of NPIs to reduce the effective reproduction number of the pathogen. As we demonstrate in Fig. 9, panel (b) of the Supplement, whilst uncertainty in the expected

effectiveness of NPIs may lead to occasional misjudgements of the optimal action that cause larger peaks in infection incidence, overall the MPC algorithm still effectively controlled the epidemic by actively reacting to the discrepancies between the actual new cases and the target. This result corroborates findings in [32] and highlights why having an adaptive strategy that considers data and action in feedback is beneficial. » Uncertainty about NPIs is an important limitation so this aspect should receive more attention. They just write in the legend of Figure S9 that the impact of NPIs is drawn from a Beta distribution but I couldn't find a clear description of the level of uncertainty used here. Essential to clarify.

We refer to our response to Question 2) detailing the issue of uncertainty in NPI efficacy. In short, we do not focus on this source of uncertainty, as we assess the pure impact of surveillance noise. However, we demonstrate that our MPC algorithm does work under NPI uncertainties as well. We now also provide clear details of the beta distribution used to model uncertainty which was indeed missing. Thanks for highlighting this issue. To make discussion and examination of uncertainty in NPI impact more central we have made the updated Fig S9 as the new Fig 7 of the main text.

11) Page 13: "While several studies have focussed on estimating and compensating for under-reporting [17] and reporting delays [47, 48, 49], these approaches often require additional knowledge about the reporting process or orthogonal data sources [50]. It is often the case that these are not available or only become available later in epidemics so we preferred to characterise performance under the more practical scenario that little else is known about the epidemic than its time series of cases." I question these statements; Yes – knowledge is imperfect, but most of the time, we can do better than just assuming that all infections are being observed; so I question the approach of the authors in not correcting for under-reporting and reporting delays.

Please see our response to Question 3). We have also revised the sentence in the manuscript by removing the phrase "more practical," to avoid implying that this is the only reasonable approach.

We agree with the reviewer that, in several recent instances, corrections for under-reporting or delays are possible and improve performance. However, such corrections typically depend on making additional assumptions or sourcing external data. These assumptions may themselves induce uncertainty and this data may be unavailable, particularly early in an epidemic. Moreover, the ability to correct for these imperfections depends strongly on location and the disease studied. For example, in some countries, surveillance is underfunded and the resulting biases are almost impossible to disentangle. Few diseases have had the resolution of monitoring that was available for COVID-19 [29] and certain types of under-reporting like asymptomatic spread are very hard to correct (even for COVID-19 there have been strongly fluctuating estimates of this effect).

Our goal in this study was not to develop or benchmark correction methods, but to explore the fundamental limits imposed by noise when only the case time series are available. While imperfect corrections can sometimes improve estimates, they can also introduce additional biases when the underlying assumptions are incorrect. The reality often lies between the extremes of perfect knowledge and naïve assumptions, both of which we cover via our performance bounds. There

are important open questions surrounding which biases are most critical to address and under what conditions. This is beyond the scope of this work but could motivate follow-up studies as the limits we exposed are the first step to gaining a wider understanding of how noise along the entire chain from data to decision impacts control performance.

12) Authors mention a projection horizon over which the assessment is performed. They mention the possibility that t_proj > t_rev, and that the algorithm assess choices made at the different decision times of this projection horizon. However, I couldn't find which value was used for t_proj (p16). Please clarify. Not clear how decision for time t+x is made at time t.

We mistakenly referred to the projection time t_proj as t_pred in Table 1, this typo is now corrected. Thank you for spotting this. We also edited the section mentioned above to better describe how the algorithm assesses action-sequences at each policy revision but only implements the first one. This offers robustness against the growing uncertainty expected from projecting further into the future. At the next policy revision, we repeat this process. We make decisions on interventions at time t based on the epidemic state that we infer from the available case data, reproduction number estimates and assumptions on the generation time. We use this information to project what the epidemic dynamics and the discounted cost function would be at t+x under each intervention option from our action space. We then choose the action minimising the costs evaluated over our projections. This is a standard setup for MPC and for reinforcement learning.

Page 7: "the average proportion of NPIs across the simulation time horizon". What is that? Please define.

The simulation time horizon is the time from the start to the end of the simulation. The average proportion of NPIs (as represented in the inset pie charts) is the average proportion of days under each NPI time used across the simulation ensemble. We include this clarification in the main text .

References

[1] S. Flaxman *et al.*, "Estimating the effects of non-pharmaceutical interventions on COVID-19 in Europe," *Nature*, vol. 584, no. 7820, pp. 257–261, Aug. 2020, doi: 10.1038/s41586-020-2405-7.

[2] J. M. Brauner *et al.*, "Inferring the effectiveness of government interventions against COVID-19," *Science (1979)*, vol. 371, no. 6531, Feb. 2021, doi: 10.1126/science.abd9338.

[3] N. Haug *et al.*, "Ranking the effectiveness of worldwide COVID-19 government interventions," *Nat Hum Behav*, vol. 4, no. 12, pp. 1303–1312, Nov. 2020, doi: 10.1038/s41562-020-01009-0.

[4] D. H. Morris, F. W. Rossine, J. B. Plotkin, and S. A. Levin, “Optimal, near-optimal, and robust epidemic control,” *Commun Phys*, vol. 4, no. 1, Apr. 2021, doi: 10.1038/s42005-021-00570-y.

[5] F. Di Lauro, I. Z. Kiss, D. Rus, and C. Della Santina, “Covid-19 and Flattening the Curve: A Feedback Control Perspective,” *IEEE Control Syst Lett*, vol. 5, no. 4, pp. 1435–1440, Oct. 2021, doi: 10.1109/lcsys.2020.3039322.

[6] K. V Parag, C. A. Donnelly, and A. E. Zarebski, “Quantifying the information in noisy epidemic curves,” *Nat Comput Sci*, vol. 2, no. 9, pp. 584–594, Sep. 2022, doi: 10.1038/s43588-022-00313-1.

[7] D. Champredon, J. Dushoff, and D. J. D. Earn, “Equivalence of the Erlang-Distributed SEIR Epidemic Model and the Renewal Equation,” *SIAM J Appl Math*, vol. 78, no. 6, pp. 3258–3278, Jan. 2018, doi: 10.1137/18m1186411.

[8] R. Anderson *et al.*, “Reproduction number (R) and growth rate (r) of the COVID-19 epidemic in the UK: Methods of estimation, data sources, causes of heterogeneity, and use as a guide in policy formulation,” 2020.

[9] P. Elliott *et al.*, “Design and Implementation of a National SARS-CoV-2 Monitoring Program in England: REACT-1 Study,” *Am J Public Health*, vol. 113, no. 5, pp. 545–554, May 2023, doi: 10.2105/ajph.2023.307230.

[10] J. Drgoňa *et al.*, “All you need to know about model predictive control for buildings,” *Annu Rev Control*, vol. 50, pp. 190–232, 2020, doi: 10.1016/j.arcontrol.2020.09.001.

[11] E. S. MEADOWS, M. A. HENSON, J. W. EATON, and J. B. RAWLINGS, “Receding horizon control and discontinuous state feedback stabilization,” *Int J Control*, vol. 62, no. 5, pp. 1217–1229, Nov. 1995, doi: 10.1080/00207179508921593.

[12] R. Dutta, S. N. Gomes, D. Kalise, and L. Pacchiardi, “Using mobility data in the design of optimal lockdown strategies for the COVID-19 pandemic,” *PLoS Comput Biol*, vol. 17, no. 8, p. e1009236, Aug. 2021, doi: 10.1371/journal.pcbi.1009236.

[13] T. Ash, A. M. Bento, D. Kaffine, A. Rao, and A. I. Bento, “Disease-economy trade-offs under alternative epidemic control strategies,” *Nat Commun*, vol. 13, no. 1, p. 3319, Jun. 2022, doi: 10.1038/s41467-022-30642-8.

[14] D. J. Haw *et al.*, “Optimizing social and economic activity while containing SARS-CoV-2 transmission using DAEDALUS,” *Nat Comput Sci*, vol. 2, no. 4, pp. 223–233, Apr. 2022, doi: 10.1038/s43588-022-00233-0.

[15] T. Britton and L. Leskelä, “Optimal Intervention Strategies for Minimizing Total Incidence During an Epidemic,” *SIAM J Appl Math*, vol. 83, no. 2, pp. 354–373, Mar. 2023, doi: 10.1137/22m1504433.

[16] K. Sherratt *et al.*, “Predictive performance of multi-model ensemble forecasts of COVID-19 across European nations,” *Elife*, vol. 12, Apr. 2023, doi: 10.7554/eLife.81916.

[17] E. L. Ray *et al.*, “Comparing trained and untrained probabilistic ensemble forecasts of COVID-19 cases and deaths in the United States,” Jan. 2022.

[18] A. Lison, S. Abbott, J. Huisman, and T. Stadler, “Generative Bayesian modeling to nowcast the effective reproduction number from line list data with missing symptom onset dates,” *PLoS Comput Biol*, vol. 20, no. 4, p. e1012021, Apr. 2024, doi: 10.1371/journal.pcbi.1012021.

[19] F. M. Shearer and M. Lipsitch, “The importance of playing the long game when it comes to pandemic surveillance,” *Proceedings of the National Academy of Sciences*, vol. 122, no. 15, Apr. 2025, doi: 10.1073/pnas.2500328122.

[20] K. Jayatilleke, “Challenges in Implementing Surveillance Tools of High-Income Countries (HICs) in Low Middle Income Countries (LMICs),” *Curr Treat Options Infect Dis*, vol. 12, no. 3, pp. 191–201, Aug. 2020, doi: 10.1007/s40506-020-00229-2.

[21] K. Charniga *et al.*, “Best practices for estimating and reporting epidemiological delay distributions of infectious diseases,” *PLoS Comput Biol*, vol. 20, no. 10, p. e1012520, Oct. 2024, doi: 10.1371/journal.pcbi.1012520.

[22] E. Conway and I. Mueller, “Joint estimation of the effective reproduction number and daily incidence in the presence of aggregated and missing data,” Jun. 07, 2024. doi: 10.1101/2024.06.06.24308584.

[23] S. W. Park *et al.*, “Estimating epidemiological delay distributions for infectious diseases,” Jan. 13, 2024. doi: 10.1101/2024.01.12.24301247.

[24] E. Goldstein, J. Dushoff, J. Ma, J. B. Plotkin, D. J. D. Earn, and M. Lipsitch, “Reconstructing influenza incidence by deconvolution of daily mortality time series,” *Proceedings of the National Academy of Sciences*, vol. 106, no. 51, pp. 21825–21829, Dec. 2009, doi: 10.1073/pnas.0902958106.

[25] S. Abbott *et al.*, “Estimating the time-varying reproduction number of SARS-CoV-2 using national and subnational case counts,” *Wellcome Open Res*, vol. 5, p. 112, Jun. 2020, doi: 10.12688/wellcomeopenres.16006.1.

[26] L. Alvarez, J.-D. Morel, and J.-M. Morel, "Modeling COVID-19 Incidence by the Renewal Equation after Removal of Administrative Bias and Noise," *Biology (Basel)*, vol. 11, no. 4, p. 540, Mar. 2022, doi: 10.3390/biology11040540.

[27] S. Bhatia, J. Wardle, R. K. Nash, P. Nouvellet, and A. Cori, "Extending EpiEstim to estimate the transmission advantage of pathogen variants in real-time: SARS-CoV-2 as a case-study," *Epidemics*, vol. 44, p. 100692, Sep. 2023, doi: 10.1016/j.epidem.2023.100692.

[28] H. J. T. Unwin *et al.*, "State-level tracking of COVID-19 in the United States," *Nat Commun*, vol. 11, no. 1, p. 6189, Dec. 2020, doi: 10.1038/s41467-020-19652-6.

[29] J. Budd *et al.*, "Digital technologies in the public-health response to COVID-19," *Nat Med*, vol. 26, no. 8, pp. 1183–1192, Aug. 2020, doi: 10.1038/s41591-020-1011-4.
